# Quasi-experimental analysis of new mining developments as a driver of deforestation in Zambia

**DOI:** 10.1038/s41598-022-22762-4

**Published:** 2022-10-29

**Authors:** Jonathan Morley, Graeme Buchanan, Edward T. A. Mitchard, Aidan Keane

**Affiliations:** 1 School of GeoSciences, University of Edinburgh, Edinburgh, UK; 2 RSPB Centre for Conservation Science, RSPB Scotland, Edinburgh, UK

**Keywords:** Environmental impact, Sustainability

## Abstract

Mining is a vital part of the global, and many national, economies. Mining also has the potential to drive extensive land cover change, including deforestation, with impacts reaching far from the mine itself. Understanding the amount of deforestation associated with mining is important for conservationists, governments, mining companies, and consumers, yet accurate quantification is rare. We applied statistical matching, a quasi-experimental methodology, along with Bayesian hierarchical generalized linear models to assess the impact on deforestation of new mining developments in Zambia from 2000 to present. Zambia is a globally significant producer of minerals and mining contributes ~ 10% of its gross domestic product and ~ 77% of its exports. Despite extensive deforestation in mining impacted land, we found no evidence that any of the 22 mines we analysed increased deforestation compared with matched control sites. The extent forest lost was therefore no different than would likely have happened without the mines being present due to other drivers of deforestation in Zambia. This suggests previous assessments based on correlative methodologies may overestimate the deforestation impact of mining. However, mining can have a range of impacts on society, biodiversity, and the local environment that are not captured by our analysis.

## Introduction

The extraction of minerals and metals through mining is key to many aspects of the global economy and society^1^. For many low- and middle-income countries, mining makes up the majority of their national gross domestic product (GDP), exports, and government revenues^2^. However, mining also involves extensive land use and can alter socio-ecological systems with impacts ranging far from the mine itself^3–5^. As much as 49.9 million km^2^ of land could be considered influenced by mining globally^6^ and it is likely an understudied contributor to deforestation in mineral-rich countries^7,8^. Given the spatial overlap of mineral deposits and areas of high biodiversity^5^, and the predicted increase in demand for mined resources over coming decades, there is potentially a significant risk to biodiversity from mining^6,9^.

Of the wide range of impacts mining can have on nearby ecosystems, deforestation is perhaps the one most clearly measurable at large spatial and temporal scales^4^. Within the mining lease itself vegetation is cleared during mineral extraction, when waste rock is dumped, and for the creation of dams to store tailings (toxic by-products of mineral extraction)^10^. Secondary infrastructure such as processing plants, buildings, and access roads, along with timber requirements for the mine, lead to further forest clearance beyond a mining lease boundary^11,12^. Additional indirect impacts include influxes of workers and their families to previously sparsely populated areas who require infrastructure such as housing, timber for building, fuel, and charcoal production, as well as land for agriculture to supplement household incomes^11–13^. These secondary impacts can often be much larger than the direct initial clearing^14^ and can happen either organically or as part of planned economic development corridors where mining and other developments are connected by linear infrastructure to open up new areas to global markets^9,15^. Mining developments can also cause the downgrading, downsizing, and degazetting of protected areas^16^, both as a consequence of a mining licence being issued and in response to increased pressure from population shifts^9,13^. An increase in forest cover is also possible in the proximity of a mine due to land management activities by the mining company as part of a restoration or compensation measures^11,17^. Understanding the deforestation footprint of mining developments is important as it represents impacts to biodiversity, through the loss of habitat^3,18^, the physical environment, through the loss of carbon and changes in ecosystem functions^19,20^, and people, through the loss of timber and non-timber forest products^21,22^.

Understanding the land cover change, especially deforestation, associated with mining is important for conservation stakeholders, governments, mining companies, and consumers higher up the supply change interested in sustainability and ecological footprints^11,23^. However, the existing literature is limited^3,23^ and most studies are correlative, meaning that deforestation within or near mining developments is measured and it is assumed that observed deforestation is attributable to mining activity^4^ and would not have occurred in the absence of mining. For example, in the Madre de Dios department of Peru, numerous studies have attributed high rates of deforestation to gold mining^24–27^. A recent study using Landsat derived land cover maps validated with very high-resolution imagery found 2,096 ha yr^−1^ (10,482 ha total) of forest loss between 2013 and 2018 within mining concessions 51% of the total deforestation for the region^24^. Previous studies in the same region of Peru had produced similar estimates of 6,145 ha yr^−1^ of forest loss between 2008 and 2012^25^ and 6,600 ha yr^−1^ between 2003 and 2009 which they attribute to new mining developments^26^. A study that utilised multiple remotely sensed datasets and covered a wider period (1985–2017) had an even greater estimate of 95,751 ha of deforestation, 64,586 ha of which happened between 2010 and 2017^27^. Elsewhere, a recent study using Landsat derived land cover maps estimated there had been 47,000 ha of deforestation across Ghana between 2005 and 2019 that can be attributed to the expansion of mining developments^28^. This follows previous, methodologically similar, studies which identified mining as the main driver of deforestation in the Asutifi North District^29^ and the Ankobrah River^30,31^. The methodology these studies use gives a measure of the direct forest loss in the vicinity of new mining developments. While this is valuable, these studies do not have a comparison to counterfactuals (what would have happened without the mines’ presence), and are therefore unable to estimate the net causal effect of mining on deforestation above the background rate^32^.

A few previous studies have used causal inference methodologies to estimate the effect of mining on deforestation separate from other drivers^4^. Butsic and colleagues used a panel instrumental variable methodology to estimate the effects of mining, armed conflicts, and protected areas on deforestation in the Democratic Republic of Congo^33^. They found mining had a significant detrimental effect on forest cover, increasing loss at a coarse spatial and temporal scale^33^. Another study of mining in India used propensity score matching at a very coarse spatial scale and found a positive association between mineral outputs at the district level and loss of forest cover with variation based on the type of minerals extracted^34^. In the Brazilian Amazon, one study used propensity score matching with post-matching regression to assess the impact of mining on deforestation in 1 km^2^ pixels^32^. They found that mining had driven extensive deforestation in the region, with deforestation being significantly higher up to 70 km from a mining lease, and estimated that 11,670 km^2^ of forest had been lost due to mining^32^. Another recent study undertook a pan-tropical analysis and found extensive forest loss directly associated with mining and a strong association between distance to mine and deforestation in the wider landscape for the majority of countries analysed^35^. However, the post-matching regression approach used in^32^ and ^35^ do not account for the spatial structure of the data and neither study tested for residual autocorrelation, suggesting that the results are potentially vulnerable to type I errors^36^ and may have inflated effect sizes^37^.

Here, we applied a quasi-experimental study design along with Bayesian hierarchical generalized linear spatiotemporal models to estimate the effect of new mining developments on deforestation in Zambia, a globally significant mineral exporter. Mining is key to the Zambian economy contributing 77% of all exports, 10% of GDP, and 29% of government revenues^38^. Zambia is the seventh largest copper producer globally and has significant industries for many other minerals and metals, including coal, precious and semi-precious stones (including 20% of the world's emeralds), and limestone^38^. Zambia is a forested country, with extensive Miombo and Mopane woodlands which are important for biodiversity conservation and the livelihoods of millions of people^22,39^. Previous analysis has concluded that mining has been a major driver of deforestation in the Copperbelt region of Zambia through both direct and indirect pathways^13^. These conclusions were however caveated by the difficulty in separating the effect of mining from other drivers^13^. We aim to build upon the work of Mwitwa and colleagues by applying a quasi-experimental methodology to isolate the effect of new large-scale mining developments from concurrent drivers of deforestation.

We identified when mining operations began in all currently issued large-scale mining leases to build a dataset of newly established mines from 2000 onwards (see Methods). We then analysed the extent of deforestation, estimated from the Hansen Global Forest Change dataset^40^, in new mining leases, and surrounding 25 km radius buffers. By including buffer areas, we aimed to capture secondary and indirect deforestation associated with mining that operate at a wider landscape scale. The buffer distance represented a conservative estimate of the range at which mines can affect nearby ecosystems^6,11^, and we tested the sensitivity of our results to this choice (See Methods). We used statistical matching methods^41,42^, with controls drawn from within large-scale exploration leases, to create a plausible counterfactual. We estimated the difference in deforestation between treated areas (i.e. leases and buffers) and matched control areas, for a period of five years after mine establishment, using statistical models to give an estimated average treatment effect. We analysed each mine individually, encompassing a range of different minerals and metals being extracted and mining processes. By analysing each mine individually our analysis can find any differences in forest lost due to different minerals and types of mining, such a surface versus underground.

Our analysis looks at deforestation both within a mining lease and a buffer, so we measure both direct and indirect forest loss. This includes land cleared to extract minerals or store tailings and the secondary effects of activities such as new roads for access, processing facilities, influxes of new workers and related development activities. Deforestation is one of a wider range of possible impacts that mining can have and therefore our study should not be taken as a comprehensive assessment of the environmental impact of mining. We consider only large-scale mining leases, not small scale or unlicenced operations. Comparison to a counterfactual means we can estimate whether new large-scale mining developments are driving deforestation at the landscape scale compared to other drivers which operate at the similar scales such as agricultural expansion, charcoal production, or urbanisation^13,21,43^. Given the existing literature we hypothesised that mining developments would have substantially increased deforestation relative to a counterfactual of no mining.

## Results

### Mines in Zambia

**Figure 1:**
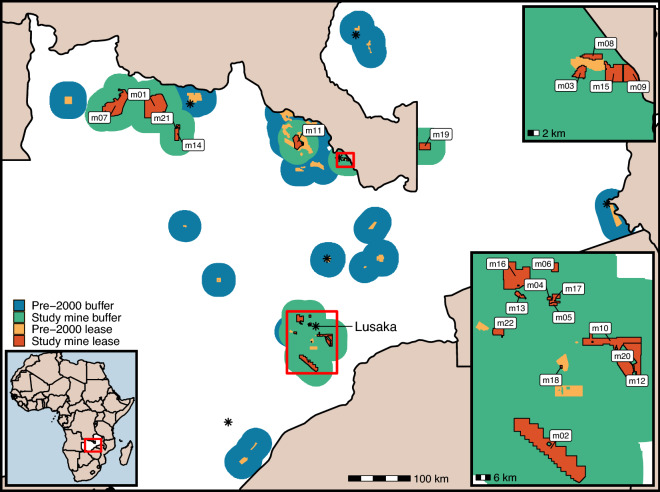
Map of large-scale mining leases in Zambia and 25 km radius buffers split by start date. Study mines leases (m01–m22) are all large-scale mining leases that started operations after 2000, the first being in 2004. Study Mine buffers are 25 km radius buffers drawn around these leases. Pre-2000 leases are large-scale mining leases which began operations before the year 2000, and pre-2000 buffers are 25 km radius buffers from these leases. Black stars are provincial capitals with the national capital Lusaka labelled. The deforestation impact of pre-2000 mines was not analysed, and the areas covered by their leases and buffers were excluded from consideration as possible controls. Where there is overlap the leases are shown, then study mine buffers, and then pre-2000 buffers. We analysed each study mine individually and considered any area falling within a lease or buffer as “treated”, ignoring overlapping buffers (i.e., treatment was coded as a binary variable). Some adjacent leases that were part of the same operation were merged. Full list of leases and details in (Sup Mat Table S1).

In total 22 mining leases were established during our study period (2000–2019) with the first new mine during this period commencing operations in 2004 (Fig. 1). This represents 3422 km^2^ of new land under a mining lease (Fig. 1), (Sup Mat Figure S1). As pre-existing leases covered an area of 2334 km^2^, the total area mined has more than doubled during the past 20 years (Sup Mat Figure S1). A further 77,868 km^2^ of land is within the 25 km buffers (Fig. 1), (Sup Mat Figure S1). The new leases were for a range of minerals with limestone being the most common followed by copper, dolomite, cobalt, and gold (Sup Mat Table S2). All but a few of the new mines were open pit surface mining (Sup Mat Table S2). The growth in mined area reflects extensive growth in the amount of copper and gold mined within Zambia (Sup Mat Figure S2).

**Figure 2:**
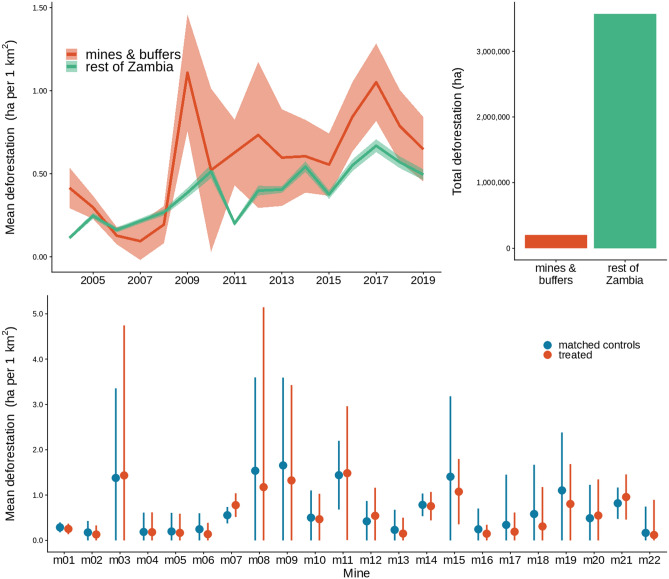
Top left panel shows the mean deforestation expressed as hectares lost per 1km^2^ pixel in Zambia estimated for all pixels which had greater than 10% tree coverage in 2000, comparing pixels within a mining lease that became active after 2000 and their 25 km buffers (red lines) and areas greater than 25 km from any mining lease (i.e., also excluding pre-existing mines and 25 km buffers; green lines). Lines are the mean and shaded areas are 1.96 times the standard error. Our deforestation metric is equivalent to tree canopy area lost for forested pixels. Top right panel shows the total deforestation in hectares for the period (2004–2019) for the same set of pixels. Bottom panel shows the mean deforestation within 1km^2^ pixels for five years after mine establishment for each mine (except mine 22 which was established only three years before 2019, the last year of deforestation data) comparing pixels within the mining lease and 25 km buffers (treated; red symbols) and matched controls (blue symbols). Points are the mean, lines are 1.96 times the standard error, with the lower bound limited to zero. These data were systematically sampled to 25% of the original data using a regular grid and filtered to only pixels which had greater than 10% tree coverage in 2000 prior to matching.

There is extensive deforestation in areas falling within new mining leases and 25 km buffers (Fig. 2). As forest pixels vary in canopy cover from 10 to 100% in Zambia, from sparse woodlands to dense forest, to ensure equivalence in terms of trees lost we used the area of tree canopy as a response variable. This means, effectively, that the clearance of a hectare with 100% canopy cover counts the same as ten hectares with 10% canopy cover. An estimated total of 201,143 ha of tree canopy area was lost, or 1,322,783 ha of total forest cover (treating all forest-covered pixels equally, regardless of canopy cover percentage, a method comparable with estimates produced by Global Forest Watch; see Methods), (Fig. 2), (Sup Mat Figure S3). There was also extensive deforestation in areas of Zambia further than 25 km from a mining lease, where a total of 3,571,373 ha of tree canopy area was cleared between 2004 and 2019 (25,679,539 ha of forest) (Fig. 2), (Sup Mat Figure S3). The rate of deforestation was on average slightly higher in new mining leases and their buffers when compared to the rest of Zambia (Fig. 2).

To estimate what the effect of mining has been on deforestation in Zambia, a plausible counterfactual (the extent of deforestation in the absence of a mine) was needed. To create a control dataset which represents this counterfactual, we used statistical matching (See Methods). A good quality match (where treatment and control are balanced in respect of key covariates) was achieved (Sup Mat Figure S4). Considering a period of five years after new mines were established, average rates of deforestation around our study mines are similar to those around matched controls (Fig. 2).

### The effect of mining on deforestation

**Figure 3:**
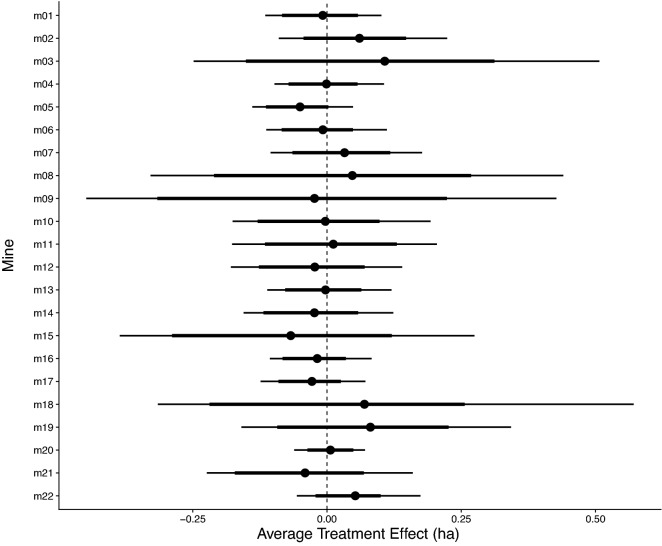
Estimated average treatment effect of mine establishment across all 5 years for each mine (except mine 22 which was established only three years before 2019 the last year of deforestation data). The effect is the difference in the area of tree canopy lost in hectares between the treatment and control areas. Estimates are from spatiotemporal models with a zero-inflated negative binomial error structure implemented in a Bayesian modelling framework using R-INLA (see Methods). Points are estimates of the mean of the posterior distribution, thick lines are the 80% credible intervals (calculated as the highest posterior density interval) and thin lines the 95%.

None of the 22 mines we analysed showed strong evidence of a treatment effect (i.e., a clear difference in tree canopy area lost between mine-affected areas and matched control areas) in the five years after mine establishment (Fig. 3). Although there were often wide credible intervals around effect estimates, the difference in deforestation for treated compared to matched control pixels was close to zero for most mines. No mine had 80% credible intervals that did not include zero (Fig. 3).

Although no overall effect was detected, the treatment effect for individual years indicates that there was elevated deforestation in treated pixels for some mines in some years. (Sup Mat Figure S5). Likely reflecting the initial large-scale clearing of vegetation for mining infrastructure, three mines (m01, m11, & m20) showed weak evidence of higher tree loss in treated pixels compared with matched control areas in the first year with 80%, but not 95%, credible intervals excluding zero (Sup Mat Figure S5). One of these mines (m20) also shows strong evidence of positive effect (i.e., greater deforestation in treated pixels) in year three, but this is balanced by strong evidence of negative effect (i.e., less deforestation in treated pixels) in years two, four, and five (Sup Mat Figure S5). Annual estimates of loss from the Hansen dataset are, however, less reliable than the five-year averages as issues with cloud cover and seasonality can lead to loss being attributed to the wrong year^44^.

We produced results for five additional datasets as sensitivity tests, varying the type of matching approach used to create the control dataset and the buffer distance. This included (1) applying coarsened exact matching, (2) using the same set-up as the main result but allowing controls to be drawn from within and outwith exploration leases, (3) a no matching, random sampling approach, (4) applying the same method as the main result but with a 50 km buffer, and (5) the same method with a 10 km buffer. The results did not change substantially with these different methods. No mines showed evidence of higher deforestation for treated pixels compared to controls under any of the matching set-ups tested (Sup Mat Figure S6). One mine showed strong evidence of lower deforestation for treated pixels when coarsened exact matching was used, and three mines estimated lower deforestation for treated pixels when using a no matching random draw of controls (Sup Mat Figure S6). Using a buffer distance of 50 km or 10 km did not substantially alter our results (Sup Mat Figure S7). One mine with a 10 and one mine with a 50 km buffer showed weak evidence of higher deforestation for treated pixels with 80% credible intervals excluding zero (Sup Mat Figure S7). The majority of models showed no residual spatial autocorrelation (Sup Mat Figure S8). We also tested the ability of our response variable, based on the Hansen dataset, to predict deforestation measured by radar remote sensing^45^. We found a weak association with very little variation between treated and control pixels (Sup Mat Figure S9).

## Discussion

We found no evidence that the 22 mines we analysed had led to excess local deforestation compared to matched control sites in the period five years after they began operations. In the five years after a new mine is established, the average rate of deforestation is not any higher than plausible counterfactual scenarios, based on matching, where no mining takes place. This was true for all mines regardless of the mineral being extracted or the extraction method. Importantly this result does not mean that there is no deforestation due to mining activity. There is clearly extensive deforestation happening within mining leases and their 25 km buffers, much of which will likely be due to direct mining activities and secondary processes, such as new roads, processing facilities, and influxes of workers, driven by the new mine being established. However, the extent of tree canopy area that is lost is no different than would likely have happened otherwise due to other drivers of deforestation in Zambia such as charcoal production, increased urbanisation, and conversion to agriculture^13,21,43^, all of which may be locally reduced due to mining activities. Zambia is a country with extensive deforestation^21,39^ and therefore the most likely counterfactual scenario is one of high rates of forest loss. Comparing deforestation in areas impacted by mining to a high background rate, in areas which are similar, results in no overall increase in tree canopy area lost.

Our results differ from two previous studies of deforestation and mining that include Zambia. Previous analysis by Mwitwa and colleagues examined deforestation in the Copperbelt region of Zambia within 50 km and 200 km of mining developments combining this with interviews of key stakeholders^13^. They found extensive deforestation within mining developments and the buffer areas and identified a number of pathways, operating at multiple spatial scales, through which mining increased deforestation^13^. Their study did not consider counterfactuals and Mwitwa and colleagues themselves note that their methodology could not separate out deforestation due to mining from other co-occurring processes such as urbanisation and the associated increased resource demands^13^. Our descriptive results are in line with their findings but once we estimate the difference from a plausible counterfactual, we do not find any evidence that that mining has driven increased deforestation and without individual mines being present much, if not all, of the deforestation would have likely taken place regardless. Another recent pan-tropical study found a strong negative association between distance to a mine and total deforestation 2000–2019 in Zambia^35^. Our results likely differ because they use proximity to any mine which is a measure of a cumulative effect of all mining as opposed to individual new mines^35^. Additionally the majority of mines included in our analysis are missing from their dataset^35^. It is also important to note that they do not account for the spatial dependence within their data nor test for spatial autocorrelation^35^ and therefore the effect they find may be inflated due to pseudo-replication.

Our results are likely to be context dependent and may not be generalizable to other regions where extensive mining takes place. In other regions mining has been found to be a driver of deforestation through secondary processes, such as new linear infrastructure, population shifts, and increases in illegal mining and logging^46^. We do not, however, find any evidence that secondary processes have caused excess deforestation. This may be because many of the new mines are surrounded by pre-existing mines, urban areas, and other intensive use land cover types. The effect of mining in these instances is likely very different compared to regions which are far from population centres with dense forest and low human population densities, as occurs in, for example, the Brazilian^32^ or Peruvian^25^ Amazon. The largest mine we examined, the Trident/Sentinel mine (Mine 7), was not near a major population centre and involved the building of several new roads, whole villages with houses, schools, and hospitals, and an airport. However this mine is unusual in that these secondary infrastructures were carefully planned and funded by the mining company along with the West Lunga Conservation Project^47,48^. These management, mitigation, and compensation measures likely reduced deforestation in the proximity of the mine, although it is important to note that nearby communities were still negatively impacted and measures which may reduce deforestation, such as restricting access to nearby forests, can have detrimental effects on food security^48^. Given these complexities we suggest that caution should be taken when generalizing studies such as this to other regions where patterns of historical land use and governance structures are different. Repetition of our approach or similar approaches that consider counterfactual rates of tree cover loss across multiple countries would contribute to our understanding of the impacts of mining on natural habitats.

Our study builds on previous studies from Zambia, but it is essential to stress that it focuses exclusively on deforestation. Mining developments can have a wide range of impacts on socio-ecological systems^3,4^. For example, contamination of water and soil with toxic chemicals is a significant problem in mining regions^49–51^. Tailings dams which store toxic by-products of mineral extraction processes in large reservoirs are a particular risk as they can fail causing pollution of soils and waterways which impacts land at some distance from the mine itself^52^. Mines can also impact socio-ecological systems, causing displacement of nearby communities and limiting their access to resources^53^. Our analysis cannot say anything about what effect the 22 newly established mines had on these outcomes. Therefore, we stress that any decisions about the issuing of new mining leases or other relevant policy decisions need to consider the full range of possible impacts.

Our analysis is limited in its ability to separate out the non-mine-specific effects and by the single level of treatment. Our methodology attempts to isolate the effect that individual mines have had on deforestation. However, it is not possible to completely separate out the broader, indirect effects that mining may have on other drivers of deforestation from the effect of specific mines being present or absent. For example, by contributing to the Zambian economy, mining is interconnected with processes such as increased urbanisation and higher resource requirements in certain regions. Additionally, we only considered a binary indicator of treatment, meaning we were not able to explore whether there were either additive or plateauing effects from a cell falling within the buffer areas of more than one mine. However, if either effect was substantial then our results would differ based on whether the mines were near other mines or not and we see no such pattern in our results.

Additional possible limitations, tested with sensitivity analysis, include the choice of buffer distance, the matching set up, and the use of the Hansen dataset as our response variable. The choice of 25 km buffers may be an underestimate of the range at which mining can impact nearby ecosystems. Other studies have assumed impacts to occur at much greater distances, for example within 50 km^6,11^, 100 km (within which significant effects were found up to 70 km)^32^, and 200 km^13^. However, the desire to capture the full extent of mine impacts must be balanced against the increased likelihood that deforestation is caused by other drivers the greater the distance from the mine, especially given Zambia’s mosaic of different land cover types. We therefore chose 25 km as a compromise and neither our overall result nor the majority of results for each mine changed when using either a 10 km or 50 km buffer. Subjective choices in application of statistical matching have been shown to potentially affect results in impact evaluations^54^. Our main results are almost completely unchanged when using coarsened exact matching, propensity score matching without the requirement that controls are drawn from exploration leases (our most restrictive condition), or a simple random draw of non-mine cells as controls. The random controls dataset showed some mines to have a negative treatment effect meaning that deforestation in these mining leases and their buffers, although similar to that under the counterfactual, was in fact lower when compared to random points in Zambia.

Our final sensitivity analysis examined whether our results were affected by the choice of forest cover data. The Hansen Forest Change dataset, which is derived from Landsat imagery, has been shown to underestimate the extent of forest loss in Zambia when compared with measures based on biomass estimated from radar remote sensing^45^. This is mainly due to its inability to see small scale losses and its sensitivity to seasonal variation in vegetation^45^. As the Hansen dataset underestimates the total change, it may also underestimate differences between mine impacted areas and our matched controls. Testing this, we found a weak but significant correlation between our response variable and an alternative data set based on radar remote sensing that covers a subset of our study period (2007–2010)^45^. There was, however, only a very small difference between treated and control pixels, with treated pixels having slightly more agreement between the two datasets. Therefore, it is unlikely that the use of the Hansen Forest Change data has substantially affected our overall result.

To conclude, our study finds no detectable effect of mines driving deforestation when compared to a matched counterfactual of no mining. This result could indicate that assessments of the deforestation impact of mining, which utilise purely descriptive or correlative methodologies, may overestimate the effect that mining has had. Statistical matching has been widely applied in other areas such as the effectiveness of protected areas^41^ but rarely used for measuring mining impacts. Our results do not mean that mining has not led to deforestation but rather we found no evidence that it was different from what would likely have happened without a mine being present. Nor does it negate the threat present to nearby ecosystems from mining, given the range of other impacts possible. Large areas of Zambia are increasingly influenced by mining activity. The extensive growth in mined land in Zambia reflects a wider trend in sub-Saharan Africa where 58% of current mines were created between 2000 and 2018, driven by growing global demand for minerals and metals^55^. This highlights the connection between land use in mineral rich nations such as Zambia and demand for natural resources globally^27,55,56^. To assess the full impact of these new mines, and other development activities, it is important that evaluations of local impact implement robust causal inference methodologies that combine statistical matching with post-match modelling that accounts for the spatial temporal nature of the data, and that they are complemented by work drawing on a broader range of quantitative and qualitative impact evaluation approaches capable of capturing the indirect effects of mining.

## Materials and methods

### Mines in Zambia

To measure the effect that new mining developments have had on deforestation, we examined 1 km^2^ pixels within Large-scale Mining Leases (LMLs) that become active from the year 2000 onwards and a 25 km radius buffer. New mining developments in Zambia are regulated through the issuance of mining leases to companies. In the first instance, exploration leases are granted that allow companies to take samples in order to estimate the presence and quantities of the resource present^38^. If minerals and metals are found in sufficient quantities to be economically viable, companies then apply for a full exploitation lease which are differentiated based on the size of the mining operation^38^. We downloaded all Large-scale Mining Leases from the Zambian government’s Mining Cadastre Map Portal (https://portals.landfolio.com/zambia/, accessed February 2020).

We assigned leases an ‘active from’ year through a multiple stage process. The date taken from the ‘Date Granted’ field in the lease dataset was first verified by visual inspection of high-resolution imagery using the time-lapse function in Google Earth pro. We looked for any evidence of the building of infrastructure or the clearing of land. If there was evidence of infrastructure being built or land cleared within two years of the granted date, this year was taken as the start date (Sup Mat Table S1). If there was no evidence to confirm this date, an internet search for industry news articles, company reports, and other documents was made. Any date suggested by these other sources was then confirmed by inspection of Google Earth imagery. If there was no evidence of activity or confirmation that the mine had not gone ahead, then the lease was marked as never active (Sup Mat Table S1). When there was uncertainty due to missing Google Earth imagery at important dates then, where possible, RGB cloud-free composites of Sentinel 2 images were inspected using Google Earth Engine. If after applying all steps the status of the mine was unclear, then it was not included in analysis and taken to be active before 2000 for the purpose of selecting controls. If multiple adjacent leases were owned by the same company and there was evidence of activity around the same time these were merged and treated as one (Sup Mat Table S1). There was a large difference for some mines between the granted date and when we found evidence of activity on site (Sup Mat Table S1).

We analysed individually all 22 mines that were found to be active after 2000, creating a dataset of treated and control units for each. Each lease was first buffered by 25 km then converted into a raster with 1 km^2^ pixels such that any cell touching the buffered lease was considered ‘treated’. We ignored cases where buffers overlap the buffers and leases of other mines. This means that in some instances pixels we considered ‘treated’ would have been previously influenced by mining by virtue of being within an existing mines buffer (see Fig. 1). All mines were treated the same regardless of the minerals being extracted or the methods of extraction, e.g., open pit versus underground shaft. Different types of mines will likely have different impacts on deforestation as they, for example, require different amounts of land to be cleared, mines of different sizes will have differing secondary impacts, and the mineral extracted will determine the amount of processing infrastructure required. By estimating the effects of each mine individually we allow for this variation.

To offer a reasonable counterfactual we considered controls drawn from Large-Scale Exploration Leases (LELs), taken from the government portal, as these are areas of land that could become treated (i.e., be converted in an active mine). All Large-Scale Mining Leases, both those included in our analysis and pre-2000 leases, and Small-scale Mining Leases buffered by 5 km, were clipped from the LELs polygons, to exclude them as potential controls. The resulting polygons were then converted to the 1 km^2^ raster with any touching pixel considered a possible control. To reduce the dataset size, and the possible effect of spatial autocorrelation, all treatment and control rasters were then subsampled to 25% of the total in a regular pattern with only the bottom right of a 4 by 4-pixel square included. These rasters were then converted to data frames of treated units and possible controls, one per mine. The datasets were further filtered to remove pixels with an average tree cover below 10%. To reduce the distance between treatment and controls the datasets were also restricted to just treatments and controls within the same province (the largest administrative unit in Zambia), determined by the province the majority of the treated units were in^57^. Due to its small size Lusaka province was merged with the neighbouring Central province. The number of treated units varied greatly depending on the size of lease, ranging from 220 to 1654 (Sup Mat Table S3). Possible controls varied based on province, ranging from 1835 to 15,979 (Sup Mat Table S3). Datasets where possible controls were drawn both within and outwith exploration leases and with a buffer distance of 50 km and 10 km were also created for sensitivity analysis (Sup Mat Table S3).

### Deforestation in Zambia

Our outcome variable of interest is deforestation expressed as the yearly area of tree canopy area lost in metres squared. Area lost and average tree cover were estimated for each 1 km^2^ pixel for each year from the Hansen Global Forest Change dataset using Google Earth Engine^40^. Yearly tree cover was calculated by converting the ‘loss year’ layer to a band per year (2001–2019), with 1 assigned to every pixel that had forest in the year 2000, with these 1s subsequently changing to 0s in a year where there was a loss event in the ‘loss year’ layer. The Hansen dataset also has a ‘tree cover’ layer, giving the percentage tree cover of each pixel in the year 2000. Zambian forests range from very sparse savannas with as little as 10% canopy cover, through to dense forests of 100% canopy cover. The latter clearly has more trees, carbon, and likely biodiversity, so we chose to weight it more highly than forest loss of low-canopy-cover forest. These yearly bands were thus multiplied by the tree cover in 2000 layer to create a multiband image with tree cover each year at the 30 m × 30 m resolution. We then converted the yearly tree covers to a 0.5 ha resolution and areas below 10% coverage at 0.5 ha were masked out to meet the Zambian government's definition of a forest^58^. Area lost was calculated by splitting the loss year layer into yearly bands, assigning the value 1 if there was loss and then converting to a 0.5 ha resolution by taking the weighted mean, giving the proportion of a 0.5 ha pixel lost each year. The 0.5 ha forest/non-forest mask for the year 2000 was then applied and the result weighted by the yearly tree cover at 0.5 ha. The resulting layers were then aggregated to the 1 km^2^ resolution using the mean, weighted by the extent of overlap, giving yearly tree cover and yearly tree canopy area lost for forested areas in 1 km^2^ pixels. The standard deviation of tree cover within a 1 km^2^ pixels (with non-forested areas masked out) was also calculated.

By weighting our area lost estimates by the tree cover in 2000 layer we create a dataset of tree canopy area lost in metres squared each year. This differs from the methodology used by Global Forest Watch^59^ and often reported by other organisations, e.g.,^21,60^. In these sources the area lost is not weighted by the extent of tree cover, meaning that a pixel with 30% tree cover being lost is treated as being the same as one with 70% tree cover. To make our results comparable with these sources we also created an unweighted dataset (which is equivalent to ‘forest area lost’, with forest defined as areas with at least 10% canopy cover over 0.5 ha) and include the relevant descriptive results in the supplementary material. One weakness of our approach is that it assumes that the tree cover in 2000 layer is a good measure of canopy cover in later years. As our method removes only pixels which appear in the loss year layer it will not capture changes in canopy cover due to regrowth and degradation not picked up as a loss by the Hansen dataset. Any changes in canopy cover due to degradation should eventually show up in the response once a threshold has been passed and regrowth will take several years to substantially impact tree canopy area at a 1 km^2^ resolution. Although our canopy area estimates may be inaccurate in later years it is reasonable to assume that the patterns of high canopy and low canopy areas will be similar, and any inaccuracies will not be biased to either treated or control pixels. Examples for two mines showing the total deforestation in the mining leases and their buffers can be seen in the supplementary material (Sup Mat Fig. 10).

The Hansen Forest Change dataset has previously been shown to underestimate forest loss in miombo woodlands^45^. To test the sensitivity of our results to this limitation we compared our deforestation measure, for pixels included in the final analysis, to estimates derived from L-Band Radar satellites. Only a limited number of years of data (2007–2010) were available^45^. We applied the same methodology to get the weighted mean of the area deforested for 1km^2^ pixels. We compared total area lost in the two datasets for the period 2007–2010 in a linear model allowing the relationship to vary for treated and control pixels (Sup Mat Fig. 9).

### Statistical matching methods

Quantitative impact evaluations estimate the effect a treatment or intervention (e.g., a mine being established) has on an outcome (deforestation) through comparison to a counterfactual, i.e., what would have been the outcome without the treatment/intervention^61^. This counterfactual does not exist and can only ever be inferred. Statistical matching is a pre-processing methodology to create a dataset of controls which represent a plausible counterfactual^62^. These controls are selected from a wider group of possible controls to be balanced in respect of key covariates to remove any bias that exists due to the non-random treatment assignment^41^. For example, mines are more likely to be established if they are near existing road networks and proximity to roads is a known predictor of deforestation. Therefore, to be plausible counterfactuals controls need to be drawn from similar distances to a road.

We assumed that deforestation was a function of the extent of forest present, ease of access, nearby populations, previous disturbance, and suitability of other land uses^32,63^. We assumed mine establishment was determined by the opportunity cost of other land uses and the ease of access. The covariates used to represent these drivers were, tree cover^40^, distance to the nearest road^64,65^, elevation and slope^66^, population density^67^, evidence of recent burning^68^, agro-ecological zone^69^, and whether a pixel was within a protected area^70^. Following best practice, we also included a measure of pre-treatment outcome, i.e., whether there had been any deforestation in the previous two years. Details of the variables and their sources are available in the supplementary material (Sup Mat Table S4). The quantity of minerals present is obviously another key driver of whether an exploration lease is converted into a full mining lease, however, as these data are not publicly available, it was not possible to include it in our analysis. As the quantities of minerals present would not impact deforestation in the absence of mining this should not substantially bias our estimates of the treatment effect.

Our primary analysis was based on a dataset generated using nearest neighbour propensity score matching (PSM). This involves estimating the probability of assignment (the propensity score) based on a logistic regression which includes the matching confounders. Treated units are then matched to the control with the nearest propensity score value. We used 1–1 matching meaning that for each treated unit a single control was included and matched without replacement so controls can only be selected once. We applied a caliper distance, so controls were only matched to the treated unit if their propensity scores are within 0.25 standard deviations of each other^42^. If no control is within this distance, then the treated unit is dropped from the dataset. We also applied a common support restriction dropping both treatment and controls units with fall outside the area of overlapping propensity scores^42^. We used exact matching for the categorical variables of the protected area and agroecological zone, meaning controls were only selected if these values were the same as the treated unit. Each mine was matched individually using confounders from the relevant year. As the matching algorithm set up has been shown to alter the overall results in impact evaluations^54^ we created three further datasets for sensitivity testing based on different matching approaches. The first used coarsened exact matching in which acceptable ranges of values are chosen for each confounder to group the data and then controls matched to treated units based on membership of the same groups for each confounder^71,72^. As many controls as match one or more treated units are included. The second used a similar matching set up as our main analysis but allowed controls to be drawn from both within and outwith exploration leases, relaxing our most restrictive matching condition. Quality of all matches was determined by examining standard mean difference plots (Sup Mat Figure S4). Our third dataset was unmatched. Instead, a random draw of controls equal to the number of treated units was selected from anywhere in Zambia (excluding all mine leases and 25 km radius buffers). After matching, the dataset size ranged between 426 and 3,306 for the main dataset and generally larger for other matching set ups (Sup Mat Table S1).

### Outcome analysis

To ensure rigorous analysis of the difference in deforestation between treated and control units, while accounting for the spatial and temporal structure of the data, we applied statistical modelling after matching. We utilised hierarchical spatiotemporal Bayesian models in which the posterior distribution is approximated through an integrated nested Laplace approximation (INLA) using the ‘R-INLA’ package in R statistical software^73,74^. The INLA methodology approximates the marginal posterior distributions of model parameters and is several orders of magnitude more computationally efficient compared to approaches that use Markov Chain Monte Carlo algorithms^75^. This computational efficiency makes it an effective means of implementing complex spatiotemporal models that would otherwise not be feasible. We utilised continuous spatial models where a spatial effect follows a Matérn covariance that is based on the solution to a stochastic partial differential equation (SPDE)^75^.

We used a spatiotemporal extension of the SPDE models to analyse deforestation each year for five years after mine establishment (except mine 22 which only had three years of data). We used an ‘exchangeable’ temporal structure in our models^76^. In these models a spatial effect is estimated for each year with values drawn from the same distribution as other years but not necessarily correlated with the previous year^76^. We included an interaction between the year and treatment effects as a fixed effect so that the treatment effect (i.e. the difference between treatment and control) is estimated for each year, and included an independent and identically distributed Gaussian random effect for the pixel ID^77^. Deforestation was rounded to the nearest whole metre square and a zero-inflated negative binomial regression used. Covariates were selected by comparing deviance information criterion values for candidate models based on the matching confounders after removing any which were collinear. Final covariates were tree cover, the standard deviation of tree cover, slope, and whether a pixel was in a protected area or not (only used for relevant mines where some pixels were within a protected area).

We used INLA’s default priors for all parameters except the hyperparameters of the spatial random field. For the spatial element we used the penalised complexity priors^76^ to control the extent of spatial smoothing with the range set to have a 95% probability of being greater than 50 km. For sigma, the prior varied between mines to ensure model convergence. Model fit was checked through posterior predictive checks and plots of simulated quantile residuals created with the ‘DHARMa’ package^78^. All direct neighbours were excluded by subsample, and we tested for residual autocorrelation at greater distances through the use of variograms. Reported results are the estimated difference in predicted loss between treated and control units for each mine, either for each year or aggregated across all years. These were created by sampling 4000 times, a number chosen to balance computing time and accuracy, from the posterior distribution using INLA’s inbuilt function.

## Supplementary Information

Supplementary Information.

## Data Availability

All code used in the analysis can be found here (https://github.com/joffy2/zam_mines_public.git) all the datasets used in analysis are publicly available, details and links are available in (Sup Mat Table S4).
