# Author Correction: Quasi-experimental analysis of new mining developments as a driver of deforestation in Zambia

**DOI:** 10.1038/s41598-023-30106-z

**Published:** 2023-03-07

**Authors:** Jonathan Morley, Graeme Buchanan, Edward T. A. Mitchard, Aidan Keane

**Affiliations:** 1 School of GeoSciences, University of Edinburgh, Edinburgh, UK; 2 RSPB Centre for Conservation Science, RSPB Scotland, Edinburgh, UK

Correction to: *Scientific Reports* 10.1038/s41598-022-22762-4, published online 29 October 2022

The original version of this Article contained errors in the reference list in the Introduction section, where References to Bini et al. (2009) and Negret et al. (2020) were omitted, and Reference 35 (Zambia EITI Council (2019)) was incorrectly given instead of Reference 43 (Giljum et al. (2022)). As a result, the References have been added and renumbered.

The omitted References are listed below.

Bini, L. M. *et al.* Coefficient shifts in geographical ecology: An empirical evaluation of spatial and non-spatial regression. *Ecography.* **32**(2), 193–204. https://doi.org/10.1111/j.1600-0587.2009.05717.x (2009).

Negret, P.J. *et al*. Effects of spatial autocorrelation and sampling design on estimates of protected area effectiveness. *Conserv. Biol.* **34**, 1452–1462. https://doi.org/10.1111/cobi.13522 (2020).

The original Article has been corrected.

