# Supplementary material for: Quasi-experimental analysis of new mining developments as a driver of deforestation in Zambia
Source: Sci Rep. 2022 Oct 29;12:18252. doi: 10.1038/s41598-022-22762-4 (PMC9617878; doi:10.1038/s41598-022-22762-4)
Supplement: Supplementary file 1 — Supplementary Information. [file 41598_2022_22762_MOESM1_ESM.pdf]

Quasi-experimental analysis of new mining developments as a driver of deforestation in Zambia

Jonathan Morley*[1], Graeme Buchanan[2], Edward T.A. Mitchard[1], Aidan Keane[1]

[1] School of GeoSciences, University of Edinburgh, Edinburgh, UK

[2] RSPB Centre for Conservation Science, RSPB Scotland, Edinburgh, UK

Corresponding author:

Jonathan Morley, jonathan.morley@ed.ac.uk, Room 401, Crew Building, West Mains Road, Edinburgh, EH9 3FF

Supplementary Material

Table S1: Details of mining leases taken from the Zambian government portal (https://portals.landfolio.com/zambia/ accessed February 2020) and the active from date they were assigned. Adjacent leases that are part of the same operation were merged. Only leases that began operations after 2000 were analysed as part of this study. Never active leases were ignored and those active before 2000 had 25 km radius buffers applied and were excluded as possible controls.

Mine	Lease code(s)	Date Granted	Active from
m01	Merged - 8089-HQ-LML, 9000-HQ-LML, 9001-HQ-LML, 9002-HQ-LML, 9003-HQ-LML, 9004-HQ-LML	06/01/2004	2004
m02	8353-HQ-LML	15/05/2006	2006
m03	22446-HQ-LML	13/03/2018	2008
m04	19456-HQ-LML	06/03/2014	2008
m05	19001-HQ-LML	16/01/2014	2008
m06	16032-HQ-LML	04/10/2011	2011
m07	Merged - 15868-HQ-LML, 15869-HQ-LML, 15870-HQ-LML, 15871-HQ-LML, 15872-HQ-LML	20/04/2011	2011
m08	Merged - 19159-HQ-LML, 22025-HQ-LML	29/08/2013	2012
m09	16773-HQ-LML	03/08/2012	2012
m10	20147-HQ-LML	20/05/2015	2013
m11	18153-HQ-LML	04/02/2013	2013
m12	18703-HQ-LML	11/08/2014	2014
m13	19437-HQ-LML	09/05/2014	2014
m14	20000-HQ-LML	16/10/2014	2014
m15	20306-HQ-LML	09/04/2015	2014
m16	20539-HQ-LML	09/07/2015	2014
m17	19725-HQ-LML	28/08/2014	2014
m18	20059-HQ-LML	23/06/2015	2014
m19	15071-HQ-LML	27/09/2012	2015
m20	20386-HQ-LML	22/04/2015	2015
m21	20501-HQ-LML	17/08/2015	2015
m22	8354-HQ-LML	22/05/2006	2017
never active	19619-HQ-LML, 20405-HQ-LML, 19255-HQ-LML, 23989-HQ-LML, 19206-HQ-LML, 19565-HQ-LML, 13837-HQ- LML, 8753-HQ-LML, 23751-HQ-LML, 12617-HQ-LML, 22447-HQ-LML		
active pre-2000	14948-HQ-LML, 7075-HQ-LML, 14532-HQ-LML, 8628-HQ-LML, 19624-HQ-LML, 17878-HQ-LML, 7064-HQ-LML, 8611-HQ-LML, 19820-HQ-LML, 7625-HQ-LML, 7065-HQ-LML, 24498-HQ-LML, 17611-HQ-LML, 7058-HQ-LML, 7057-HQ-LML, 20813-HQ-SML, 20282-HQ-LML, 12848-HQ-LML, 25089-HQ-LML, 14182-HQ-LML, 7069-HQ-LML, 17526-HQ-LEL, 8752-HQ-LML, 8750-HQ-LML, 12634-HQ-LML, 20584-HQ-LML, 8625-HQ-LML, 7071-HQ-LML, 8245-HQ-LML, 17868-HQ-LML, 17894-HQ-LML, 7045-HQ-LML, 13207-HQ- LML, 13287-HQ-LML, 7061-HQ-LML, 20809-HQ-LML, 15547-HQ-LML, 6990-HQ-LML, 21816-HQ-LML, 8097-HQ-LML, 7076-HQ-LML, 7074-HQ-LML, 7073-HQ-LML, 8622-HQ-LML, 12985-HQ-LML, 13881-HQ-LML, 8509-HQ-LML, 8323-HQ-LML, 8325-HQ-LML, 8749-HQ-LML, 8392-HQ-LML, 19740-HQ-LML, 8748-HQ-LML, 8474-HQ-LML, 8404-HQ-LML, 8403-HQ-LML, 8403-HQ-LML, 8396-HQ-LML, 8395-HQ-LML, 8394-HQ-LML, 8393-HQ-LML, 17164-HQ-LML, 13880-HQ-LML		

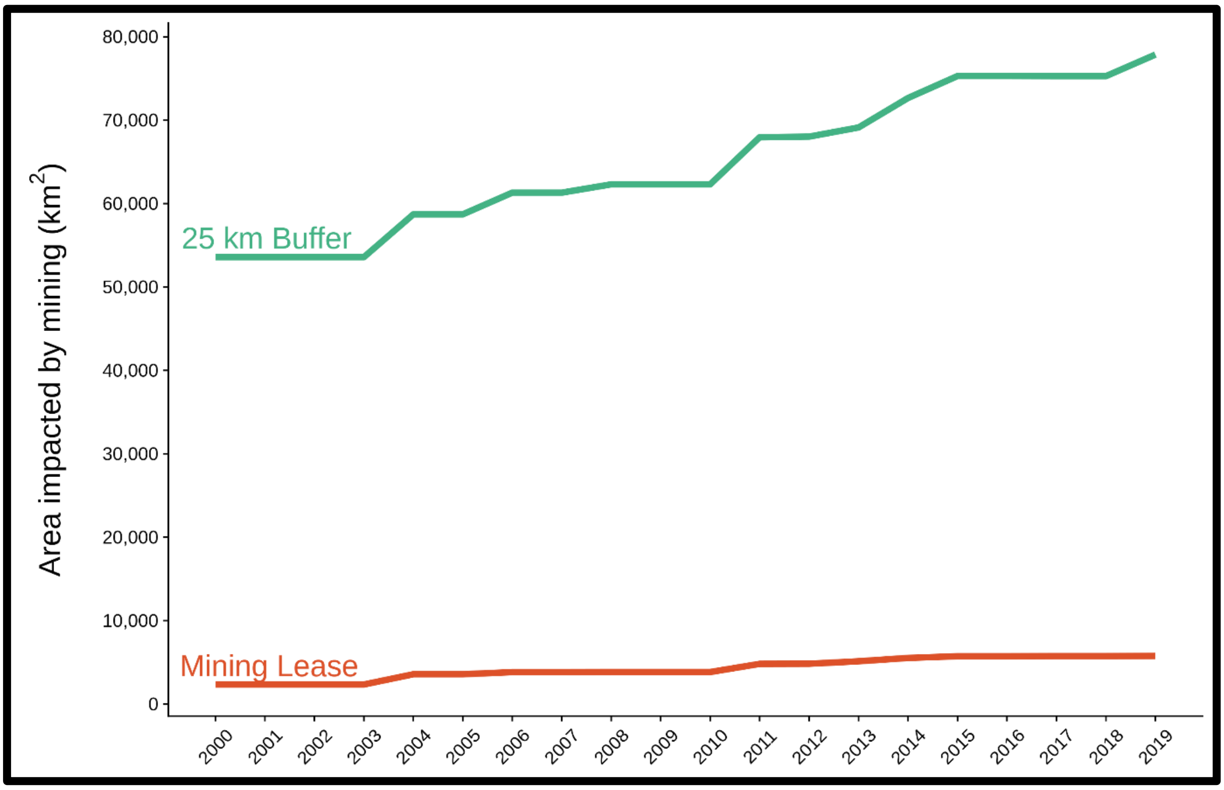

Figure S1: Growth in mine impacted area over time from 2000 onwards separated by pixels within mining leases and 25 km radius buffers.

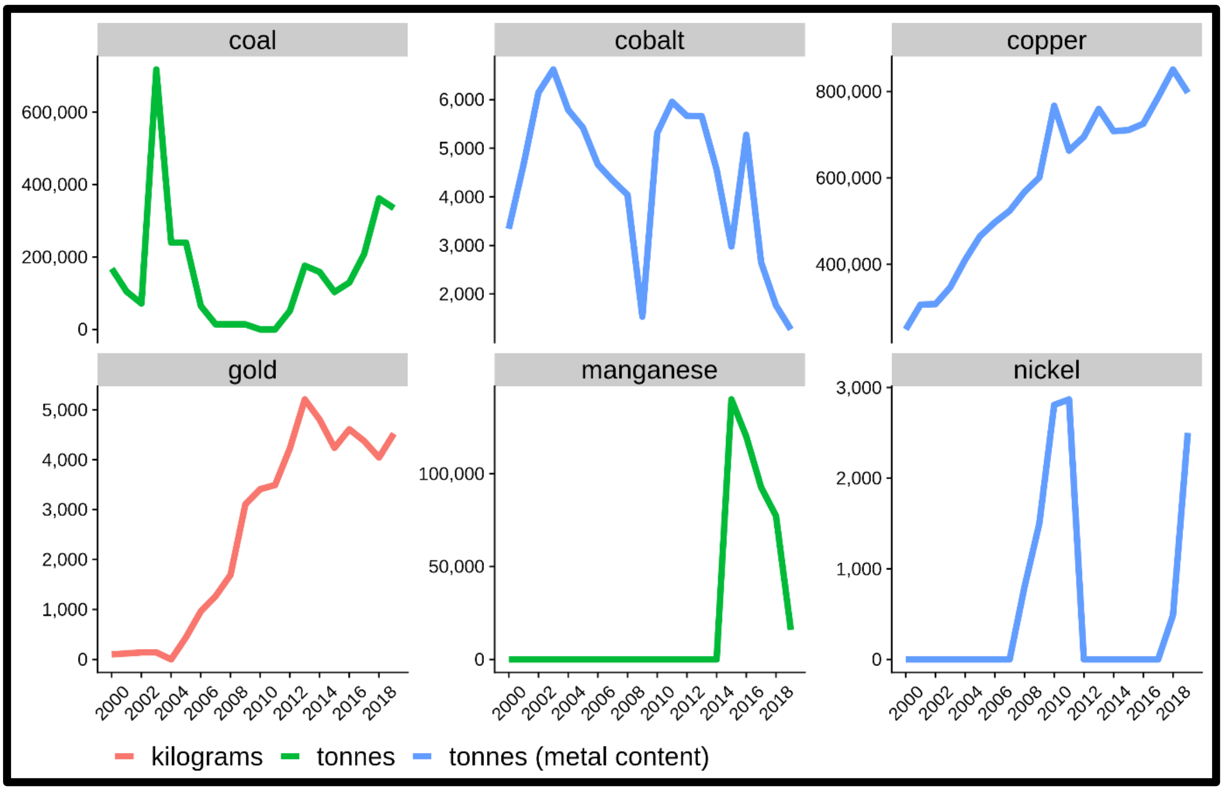

Figure S2: Growth in production of selected mined resources 2000 to 2019 in Zambia. Data is taken from (https://www2.bgs.ac.uk/mineralsuk/). Minerals shown represent the major resources in Zambia and are extracted in one or more of our study mines (Table S2). The different colours represent different units of measurement. Zambia also produces substantial amounts of emeralds, other gemstones, and limestone. These are absent from the figure as comparable data is not available.

Table S2: Commodities that are licenced for extraction at the mining leases of the 22 study mines. Taken from the Zambian government portal (https://portals.landfolio.com/zambia/ accessed February 2020).

mine	Commodities	Extraction method
m01	Cobalt, Copper, Gold, Silver, Sulfur, Uranium	Open pit
m02	Bismuth, Chromium, Coal, Cobalt, Copper, Diamond, Gold, Iron Ore, Lead, Manganese, Molybdenum, Nickel, Phosphorus-Phosphates, Selenium, Silver, Sulfur, Tellurium, Thorium, Titanium, Uranium, Vanadium, Zinc, Zirconium	Underground
m03	Dolomite, Laterite, Limestone (General)	Open pit
m04	Dolomite	Open pit
m05	Limestone (General), Stone	Open pit
m06	Crushed/Broken Stone, Dolomite, Limestone (General)	Open pit
m07	Cobalt, Copper, Gold, Iron Ore, Nickel, Platinum Group Metals, Selenium, Silver	Open pit
m08	Limestone (General)	Open pit
m09	Limestone (General)	Open pit
m10	Dolomite, Limestone (General)	Open pit
m11	Copper	Open pit
m12	Limestone (General)	Open pit
m13	Dolomite, Granite, Limestone (General)	Open pit
m14	Cobalt, Copper, Gold, Granite, Limestone (General), Manganese	Unclear
m15	Limestone (General)	Open pit
m16	Limestone (General)	Unclear
m17	Limestone (General), Marble	Open pit
m18	Dolomite, Granite, Limestone (General)	Open pit
m19	Amethyst, Cobalt, Copper, Lead, Manganese, Quartz, Tin, Tourmaline, Zinc	Unclear
m20	Dolomite, Limestone (General)	Open pit
m21	Cobalt, Copper, Gold, Iron Ore, Limestone (General), Manganese, Silver, Zinc	Open pit
m22	Copper, Gold, Iron Ore, Limestone (General), Manganese, Nickel, Stone	Mountaintop and open pit

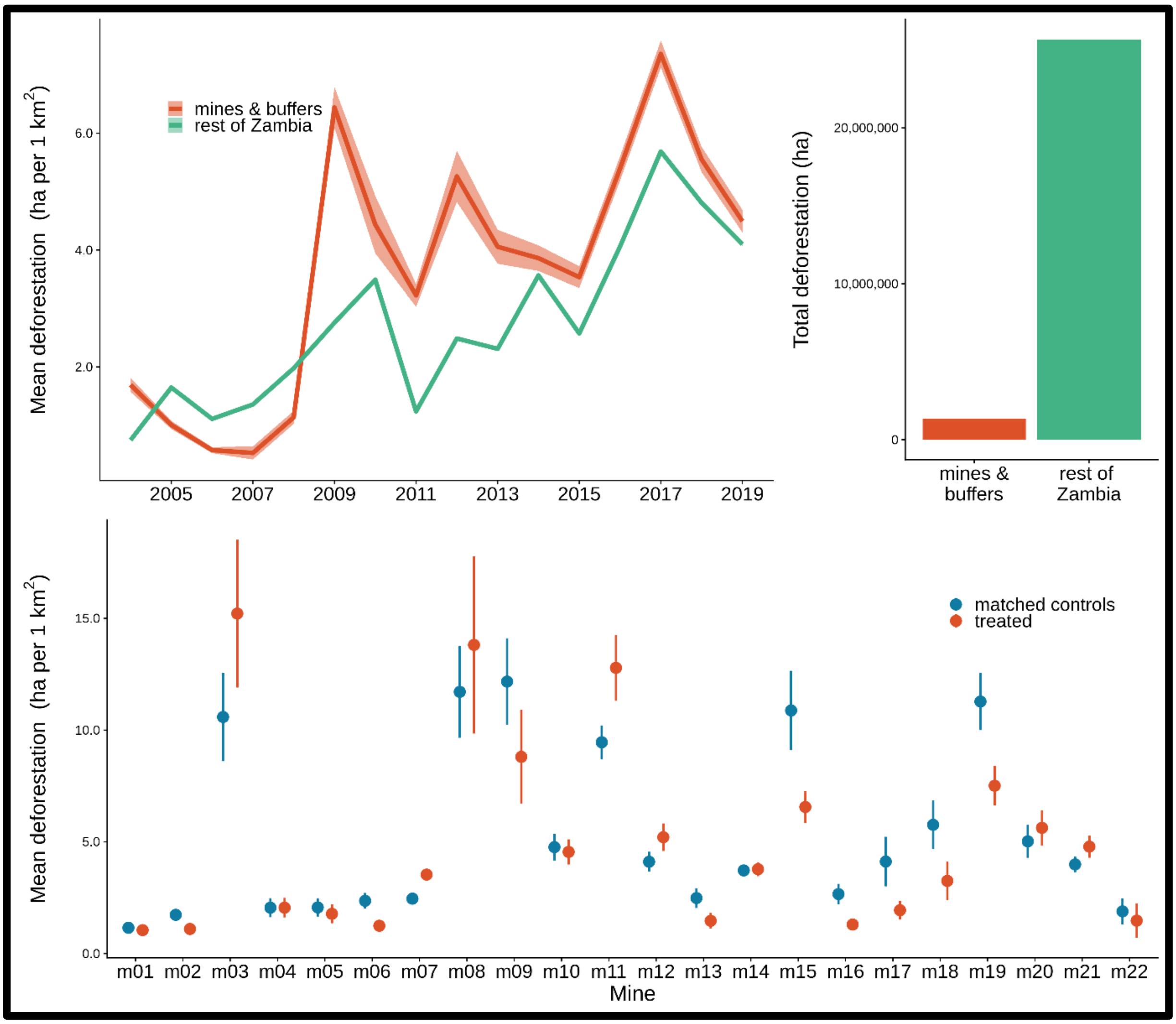

Figure S3: This figure is identical to Figure 2 in the main text but using values for deforestation which are unweighted by tree cover to be comparable to other sources (See Methods).

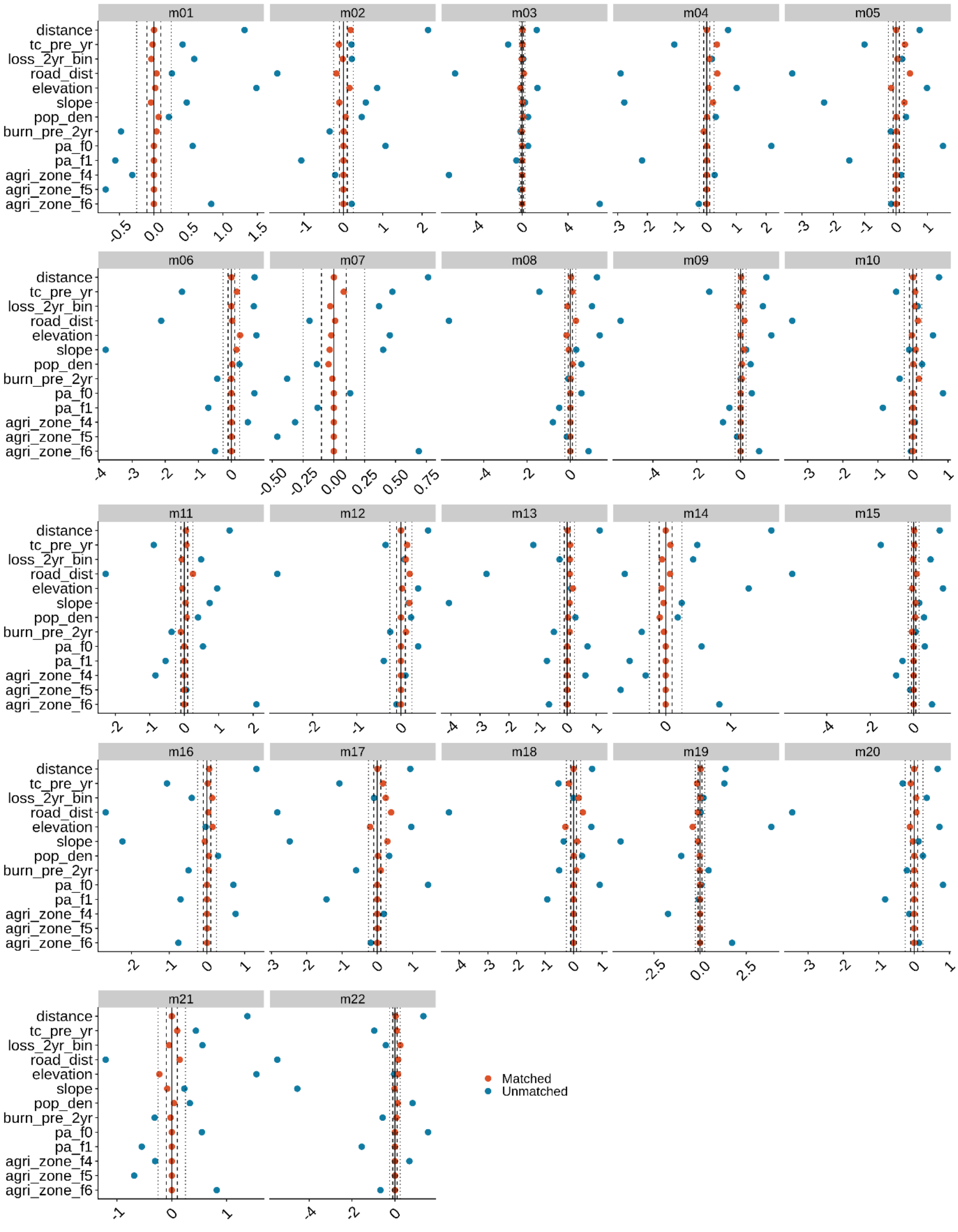

Figure S4: Standardized mean difference plot for main dataset comparing difference between treated and control pixels for key covariates in the dataset before matching and after applying propensity score matching. Distance is the Propensity Score, i.e., the probability of being treated. *Tc_pre_yr* is the tree cover in year prior to mine being established; *loss_2yr_bin* is a binary value of whether there had been any deforestation in the previous two years; *road_dist* is distance to the nearest road; *pop_den* is the density of human population; *burn_pre_2yr* is number of months with a burning event in the previous two years; *pa_f* variables indicates whether the pixel is within a protected area, *agri_zone* variables indicate which agro-ecological zone a pixel is a member of.

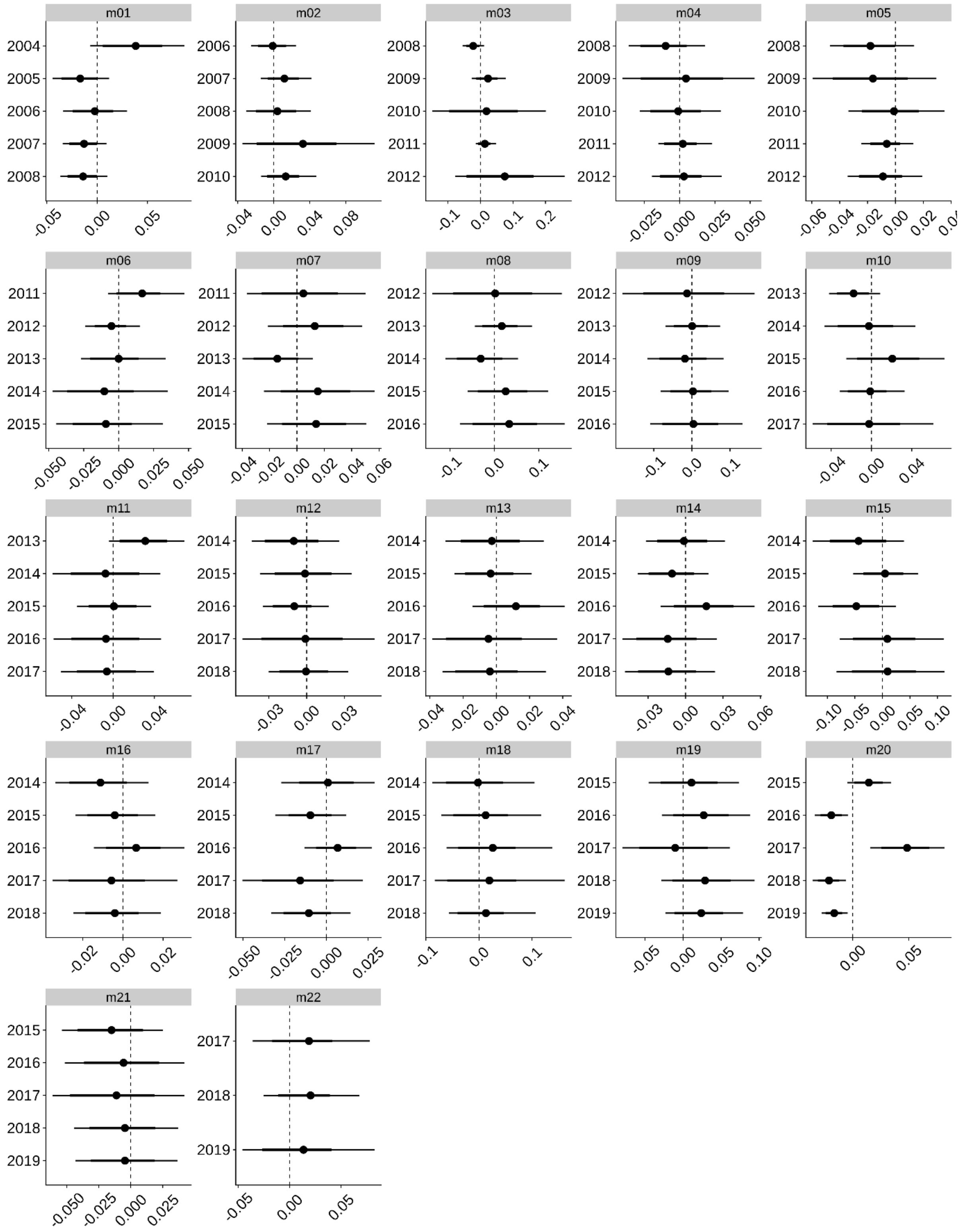

Figure S5: Average treatment effect (difference between treatment and control groups) for each year. Values are differences in area of tree canopy lost in hectares. Estimates are for each year for 5 years after a mine is established. Estimates are from spatial-temporal models with a zero-inflated negative binomial error structure implemented in a Bayesian modelling framework using R-INLA. Points are estimates of the mean of the posterior distribution, thick lines are the 80% credible intervals (calculated as the highest posterior density), and thin lines are the 95% credible intervals.

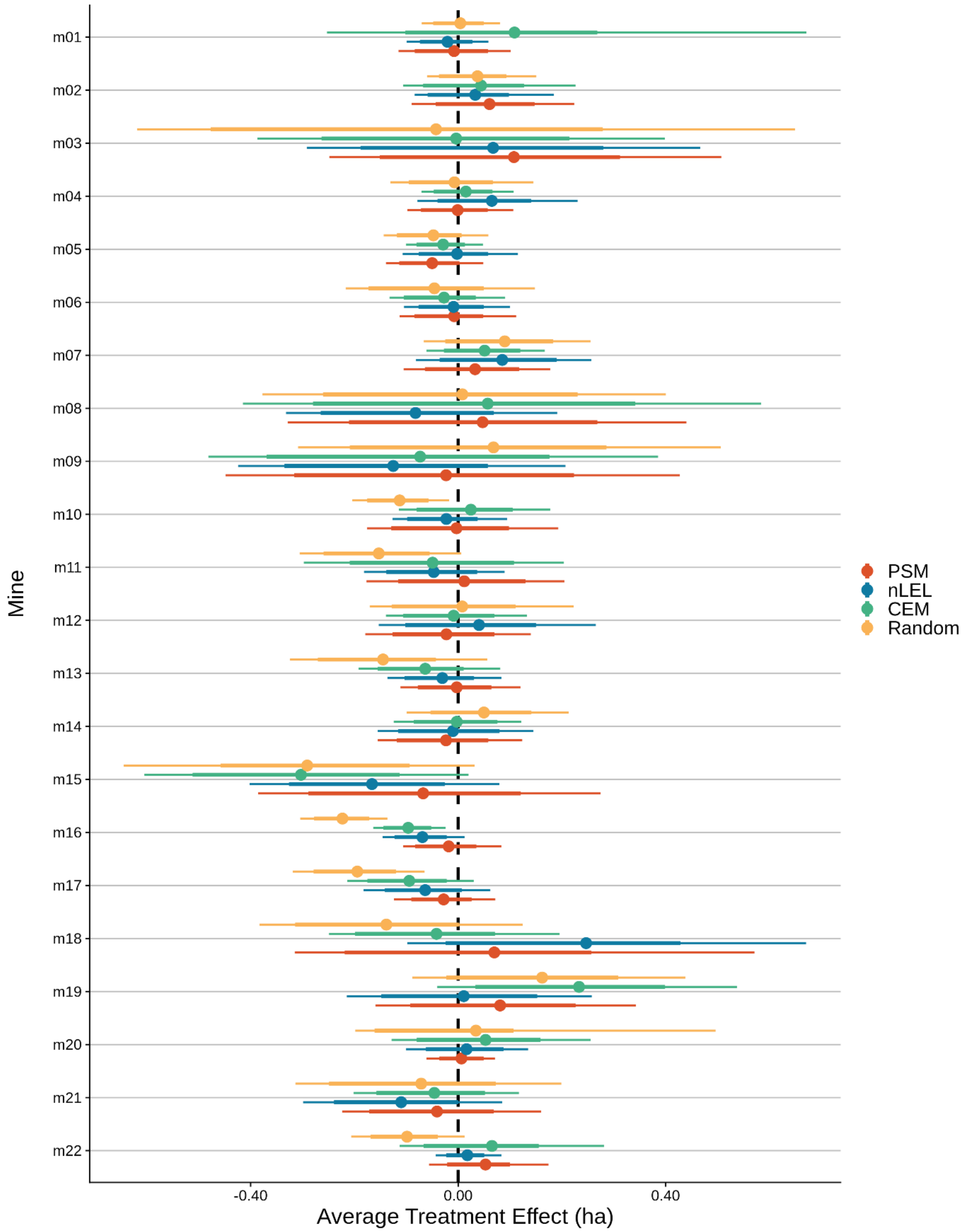

Figure S6: Aggregate five-year average treatment effect for each mine comparing different matching methods. PSM is Propensity Score Matching and is the dataset reported as the main result. CEM is Coarsened exact Matching, nLEL is the same as PSM but controls are drawn from both within and outwith Large-scale Exploration Leases, Random is a dataset with a random draw of controls equal to the number of treated cells. Estimates are from spatial-temporal models with a zero-inflated negative binomial error structure implemented in a Bayesian modelling framework using R-INLA. Points are estimates of the mean of the posterior distribution, thick lines are the 80% credible intervals, calculated as the highest posterior density, and thin lines are the 95% credible intervals.

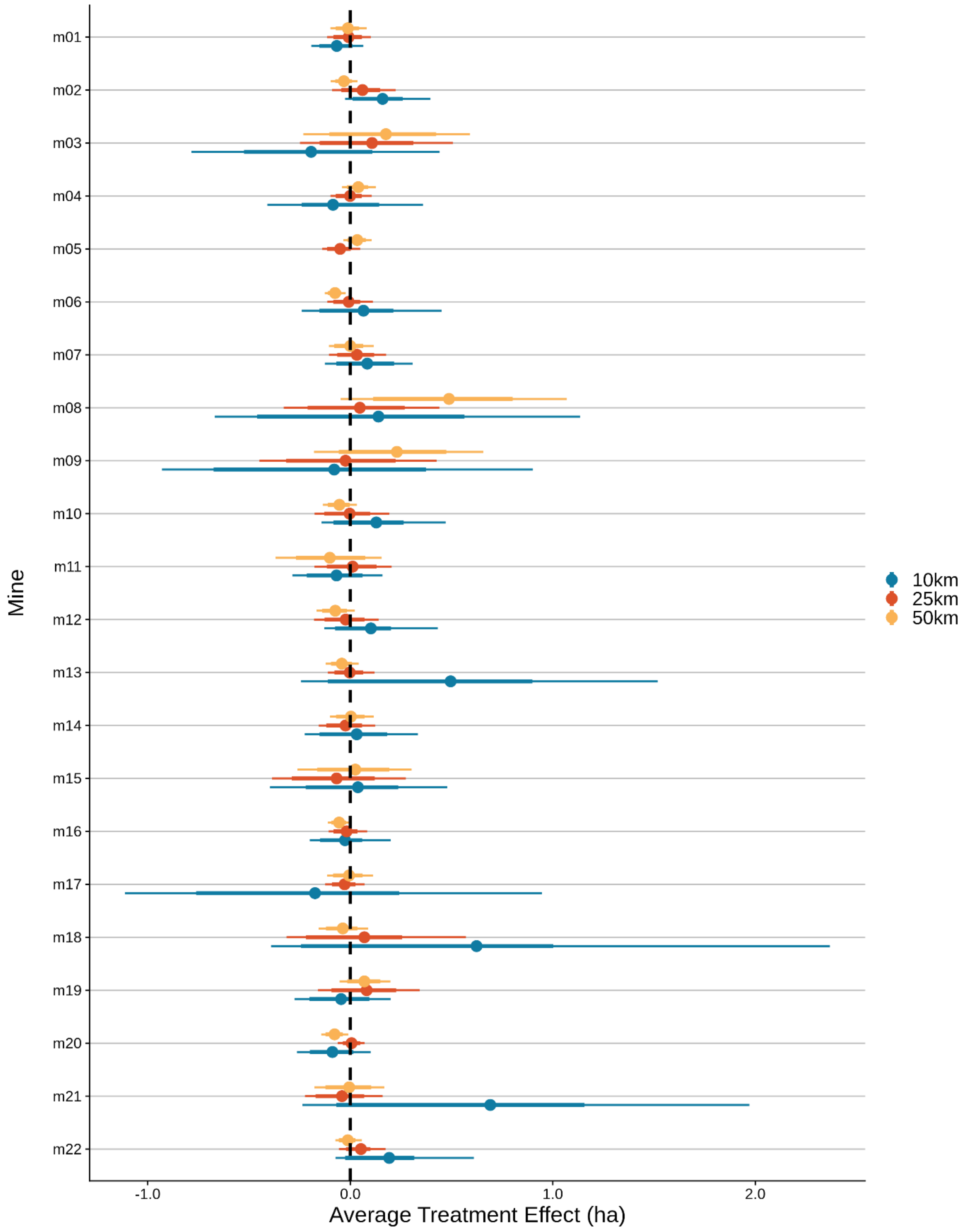

Figure S7: Aggerate five-year average treatment effect for each mine comparing different buffer distances. Dataset reported in main results is 25 km. Model for Mine 5 in the 10 km buffer dataset did not converge. Estimates are from spatial-temporal models with a zero-inflated negative binomial error structure implemented in a Bayesian modelling framework using R-INLA. Points are estimates of the mean of the posterior distribution, thick lines are the 80% credible intervals, calculated as the highest posterior density, and thin lines are the 95% credible intervals.

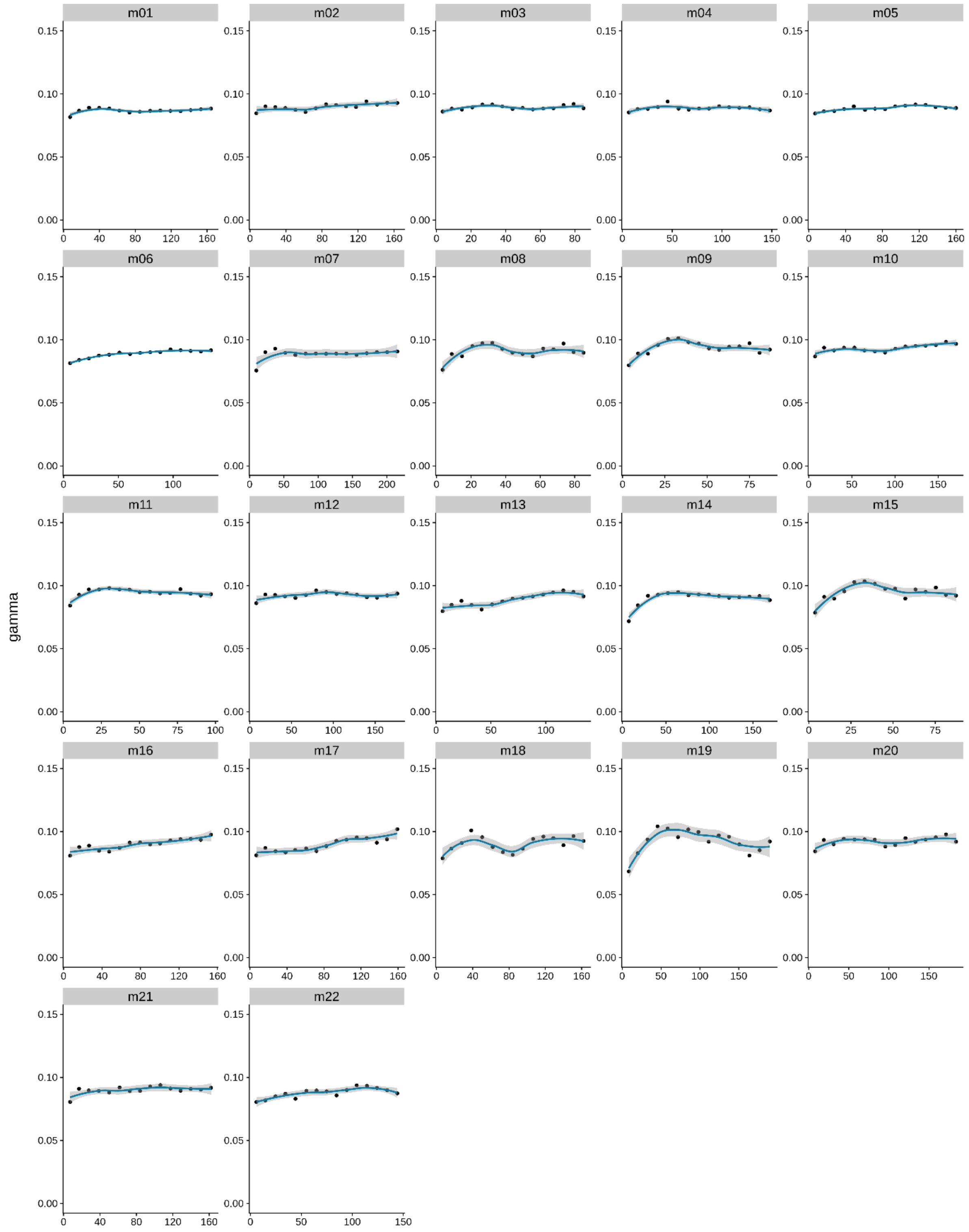

Figure S8: Variograms of simulated scaled quantile residuals estimated with the DHARMa package for each model. Plots show the change in variance for groups of points at increasing distances from each other. If there is no spatial dependency in the residuals, then the variance should not increase with distance and the lines should be approximately flat. This is not the case for some mines. However, as the datasets were sub-sampled to remove direct neighbours (i.e., no pixels are within 1.5 km of each other) and the presence of spatial autocorrelation tends to reduce standard errors and inflate effect sizes the residual spatial dependency are unlikely to be strongly influencing the interpretation of the results, i.e., that there is no evidence of a positive treatment effect.

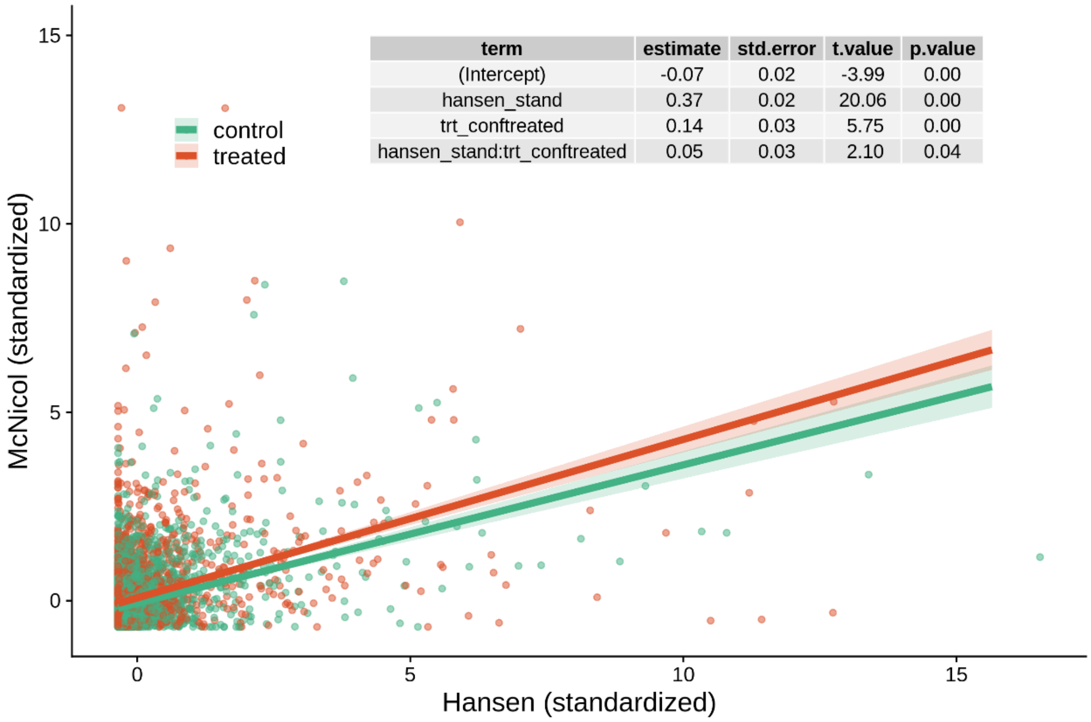

term	estimate	std.error	t.value	p.value
(Intercept)	-0.07	0.02	-3.99	0.00
hansen_stand	0.37	0.02	20.06	0.00
trt_conftreated	0.14	0.03	5.75	0.00
hansen_stand:trt_conftreated	0.05	0.03	2.10	0.04

Figure S9: Data and predictions from a linear model where our Hansen-based response predicts deforestation estimated from radar remote sensing. The model was fit in a frequentist framework with an interaction between treatment status and the Hansen-based response. The inset table shows the estimates of model coefficients effects and lines are the slopes for treated and control units with confidence intervals. The points are the data used in the model. There is a very small difference between treated and control pixels with the Hansen response in treated pixels being slightly better at predicting the radar-based response.

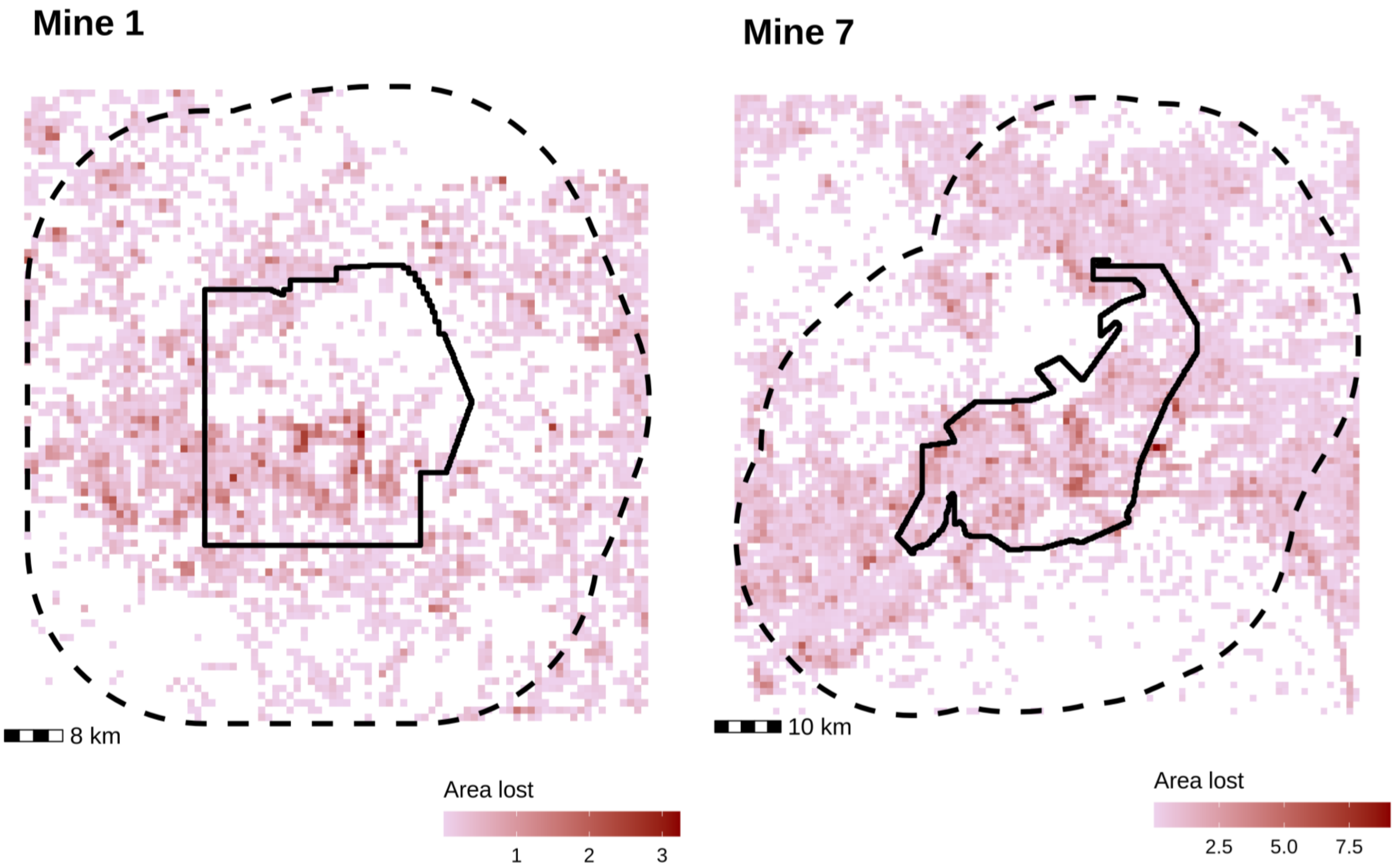

Figure S10: Example mines (mine 1 left and mine 7 right) showing the total deforestation in the period 5 years after mine establishment measure. Value is hectares of tree canopy area lost within each 1 km pixel. Both the largescale mining lease (solid black line, and 25 km buffer (dashed line) are shown. Values are calculated from Hansen dataset (see Methods), raw data can be seen at global forest watch website https://www.globalforestwatch.org/map/)

Table S3: Dataset size for each mine before and after each matching is applied. These are the values for 25% subsampled dataset. PSM Match is Propensity Score Matching, the dataset reported as the main results. CEM is coarsened exact matching a different matching method. nLEL is the same as PSM but controls come from both within and outwith Large-Scale Exploration leases. These values are for a single year, deforestation was analysed for five years (three for mine 22) so *n* for statistical models are these values multiplied by the number of years.

	Unmatched data			PSM match		CEM match		nLEL PSM match	
**Mine**	**Treated**	**LEL controls**	**nLEL controls**	**Treated**	**Controls**	**Treated**	**Controls**	**Treated**	**Controls**
m01	1,583	15,979	26,582	1,583	1,583	1,565	6,024	1,583	1,583
m02	751	1,835	10,646	464	464	315	407	748	748
m03	279	3,345	6,752	235	235	232	451	275	275
m04	305	9,460	21,074	255	255	223	1,746	263	263
m05	318	9,460	21,074	254	254	230	1,825	271	271
m06	347	9,460	21,074	316	316	286	2,358	325	325
m07	1,654	15,979	26,582	1,653	1,653	1,625	8,840	1,652	1,652
m08	237	3,345	6,752	190	190	195	515	230	230
m09	220	3,345	6,752	199	199	180	479	211	211
m10	570	9,460	21,074	485	485	399	2,386	516	516
m11	811	3,345	6,752	671	671	664	1,361	787	787
m12	510	9,460	21,074	455	455	345	2,412	473	473
m13	392	9,460	21,074	350	350	304	1,448	372	372
m14	914	15,979	26,582	914	914	896	4,687	914	914
m15	243	3,345	6,752	213	213	193	436	233	233
m16	570	9,460	21,074	511	511	446	1,640	557	557
m17	350	9,460	21,074	266	266	255	1,557	288	288
m18	220	9,460	21,074	180	180	136	1,174	191	191
m19	629	9,460	21,074	595	595	493	989	621	621
m20	359	9,460	21,074	315	315	245	2,054	326	326
m21	514	15,979	26,582	514	514	509	4,086	514	514
m22	309	9,460	21,074	234	234	229	980	292	292

Table S4: Confounders and other data used in analysis and their source

Variable	Range	Details	Link to source
Mining leases		Large scale Mining Leases. Accessed February 2020	https://portals.landfolio.com/zambia
Tree cover	Each year 2001-2020	Percentage tree canopy cover in 1 km^2 pixel for the previous year. Estimated from Hansen Global Forest Change v1.7 dataset.<br><br>Non-forest areas masked. Yearly value calculated by removing deforested pixels. Previous year used to be cover before mine established.	earthenginepartners.appspot.com/science-2013-global-forest
Deforestation	Each year 2001-2019	Area of tree canopy cover in a 1km^2 pixel lost each year in m^2. Non-forest pixels, defined as areas with <10% canopy cover at 0.5 ha resolution, removed - this definition comes from Zambia's national definition.	As above
Pre-treatment outcome	Each year 2002-2020	Binary presence or not of any deforestation in previous two years.	As above
Distance to nearest road	2000-2009<br><br>2010-2020	Distance to nearest 1 km^2 pixel containing a road. Roads are taken from two datasets as to ensure value is from before treatment date. Values are constant within the two time periods.	Pre-2010 roads are from: https://gis-lab.info/qa/vmap0-eng.html<br><br>2010 onwards from: https://sedac.ciesin.columbia.edu/data/set/groads-global-roads-open-access-v1/maps?facets=region:africa
Elevation and slope	Constant	The weighted mean elevation and slope within a 1km2 pixel estimated from the SRTM Digital Elevation Data Version 4 dataset using inbuilt functions from Google Earth Engine. Slope was estimate at original resolution (90 by 90 m).	https://srtm.csi.cgiar.org
Population	Each year 2000-2020	Population density in a 1 km^2 pixel Worldpop yearly population datasets adjusted for UN country totals	https://www.worldpop.org/doi/10.5258/SOTON/WP00675

Previous burning	Each year 2002-2020	Number of months there was burning within a pixel in the previous two years. Estimated from MCD64A1.006 MODIS Burned Area Monthly Global.	https://lpdaac.usgs.gov/products/mcd64a1v006
Protected area	Constant	A binary value whether a pixel in within a National Park, Game Management Area or Forest Reserve. Data taken from the World Database on Protected Areas, accessed February 2020.	https://www.protectedplanet.net/en
Agroecological zone	Constant	Which agroecological zone a pixel is in taken from CELL5M: A Multidisciplinary Geospatial Database for Africa South of the Sahara.	https://dataverse.harvard.edu/dataset.xhtml?persistentId=doi:10.7910/DVN/G4TBLF
Zambian provinces	Constant	Country and province outlines from GADM database v3.4	https://gadm.org/data.html
